# Trends in knee osteoarthritis prevalence over a 10-year period in Japan: The ROAD study 2005–2015

**DOI:** 10.1016/j.ocarto.2025.100569

**Published:** 2025-01-25

**Authors:** Gaku Tanegashima, Toshiko Iidaka, Shigeyuki Muraki, Chiaki Horii, Hiroyuki Oka, Hiroshi Kawaguchi, Kozo Nakamura, Toru Akune, Sakae Tanaka, Noriko Yoshimura

**Affiliations:** a Department of Orthopaedic Surgery, Sensory and Motor System Medicine, Graduate School of Medicine, The University of Tokyo, Tokyo, 113-8655, Japan; b Department of Prevention Medicine for Locomotive Organ Disorders, 22nd Century Medical and Research Center, The University of Tokyo, Tokyo, 113-8655, Japan; c Division of Musculoskeletal AI System Development, Faculty of Medicine, The University of Tokyo, Tokyo, 113-8655, Japan; d Nadogaya Hospital, Chiba, 277-0084, Japan; e Towa Hospital, Tokyo, 120-0003, Japan; f National Rehabilitation Center for Persons with Disabilities, Saitama, 359-0042, Japan

**Keywords:** Osteoarthritis, Knee, Prevalence, Cohort study

## Abstract

**Objective:**

This study aimed to clarify the trends in the prevalence of knee osteoarthritis (OA) and symptomatic knee OA among the general population using population-based cohort data from baseline and a survey 10 years later.

**Design:**

The baseline survey of the Research on Osteoarthritis/Osteoporosis Against Disability (ROAD) study was conducted from 2005 to 2007; 3040 participants (1061 men and 1979 women; mean age 70.3 years) completed all OA examinations, including a questionnaire of medical information in the present/past and radiographic examination. The fourth survey was performed from 2015 to 2016; 2893 individuals (895 men and 1998 women, mean age 68.9 years) completed assessments identical to those at the baseline survey. Knee OA was defined using the Kellgren–Lawrence grading system.

**Results:**

The prevalence of knee OA was 54.6 ​% (men, 42.0 ​%; women, 61.5 ​%) at the baseline survey and 39.3 ​% (men, 26.9 ​%; women, 44.9 ​%) at the fourth survey, with a significant decrease (p ​< ​0.0001). The prevalence of symptomatic knee OA was 24.3 ​% (men, 16.9 ​%; women, 28.3 ​%) at the baseline survey and 20.6 ​% (men, 14.2 ​%; women, 23.5 ​%) at the fourth survey, showing a similar decrease (p ​< ​0.0001). Thus, the prevalence of knee OA and symptomatic knee OA was lower at the fourth survey than at the baseline survey (p ​< ​0.01).

**Conclusions:**

In the population-based survey with a 10-year interval, the prevalence of knee OA and symptomatic knee OA decreased significantly. This preferable change in OA may suggest rejuvenation in the current population and could contribute to a decrease in the occurrence of disabilities in the future.

## 1 Introduction

Knee osteoarthritis (OA) has caused chronic pain and disability in many developed countries over the past few decades [[1], [2], [3]]. It is characterised by pathological and radiographic features, including joint space narrowing and osteophytosis. Progression is irreversible and leads to catastrophic disability, which directly lowers the patients’ quality of life [4]. According to the Ministry of Health, Labor and Welfare, the latest survey shows an increase in the osteoarthritis group, which landed fifth (10.2 ​%) in the overall cause of nursing care. However, this was first observed in individuals requiring support (19.3 ​%) [5]. There is an even greater need for strategies to prevent and treat this condition.

In 2005, we established a large-scale population-based OA cohort study called the Research on Osteoarthritis/Osteoporosis Against Disability (ROAD) with the goal of establishing epidemiologic indices to evaluate clinical evidence for the development of a disease-modifying treatment for skeletal diseases [6,7]. We created a baseline database with detailed clinical and genetic information on three population-based cohorts in the urban, mountainous, and coastal communities of Japan. Using the data acquired from the ROAD study, we reported the prevalence of osteoporosis and hip OA [7,8]. Regarding knee OA and symptomatic knee OA, we clarified the prevalence, incidence, and risk factors of knee OA among older adults (≥60 years), along with its association with pain [9,10]. These compiled data were utilised to launch governmental assets for the prevention of osteoporosis, knee OA, and hip OA.

However, in 2015, 10 years after the initiation of the ROAD study, an update of the estimated number of individuals with knee OA was required. Moreover, interpreting long-term changes in radiographic and symptomatic knee OA is an outstanding tool for gathering epidemiological data, which has not been established in Japan. These data can also help develop strategies to prevent knee OA. Thus, we conducted a fourth survey in 2015 and 2016 to evaluate the differences over a 10-year period. Using the data acquired from the fourth survey, we previously elucidated that the prevalence of osteoporosis and hip OA had decreased in the younger generation over 10 years [7,8]. However, the prevalence of knee OA and symptomatic knee OA after 10 years later and its trends remain unknown.

This study aimed to reveal trends in the prevalence and distribution of knee OA and symptomatic knee OA using cohorts from the ROAD study at the baseline survey and the fourth survey, which was conducted 10 years after the initial survey.

## 2 Method

### 2.1 Participants

The ROAD study was a prospective investigation of OA conducted among population-based cohorts in various cities across Japan. The survey selected three distinct residential areas: Itabashi (urban) in Tokyo, Hidakagawa (mountainous), and Taiji (coastal) in Wakayama Prefecture. We confirmed that each residential area had a background similar to that of Japan's general population [6,7,9]. We initially enrolled 3040 participants (1061 men and 1979 women; mean age 70.3 years; range 23–95 years) during the baseline period and have since conducted multiple follow-up surveys since 2005. At baseline, the participants were recruited from resident registration listings in three communities with different characteristics: 1350 participants from an urban region in Itabashi, Tokyo; 864 participants from a mountainous region in Hidakagawa, Wakayama; and 826 participants from a coastal region in Taiji, Wakayama. Participants who could walk to the survey site, report data, and understand/sign the informed consent form were included. These included baseline (2005–2007, 3040 participants) and fourth (2015–2016, 2893 participants) surveys. Each follow-up survey aimed to reengage participants from previous surveys and recruit new participants from each area.

**Fig. 1:**
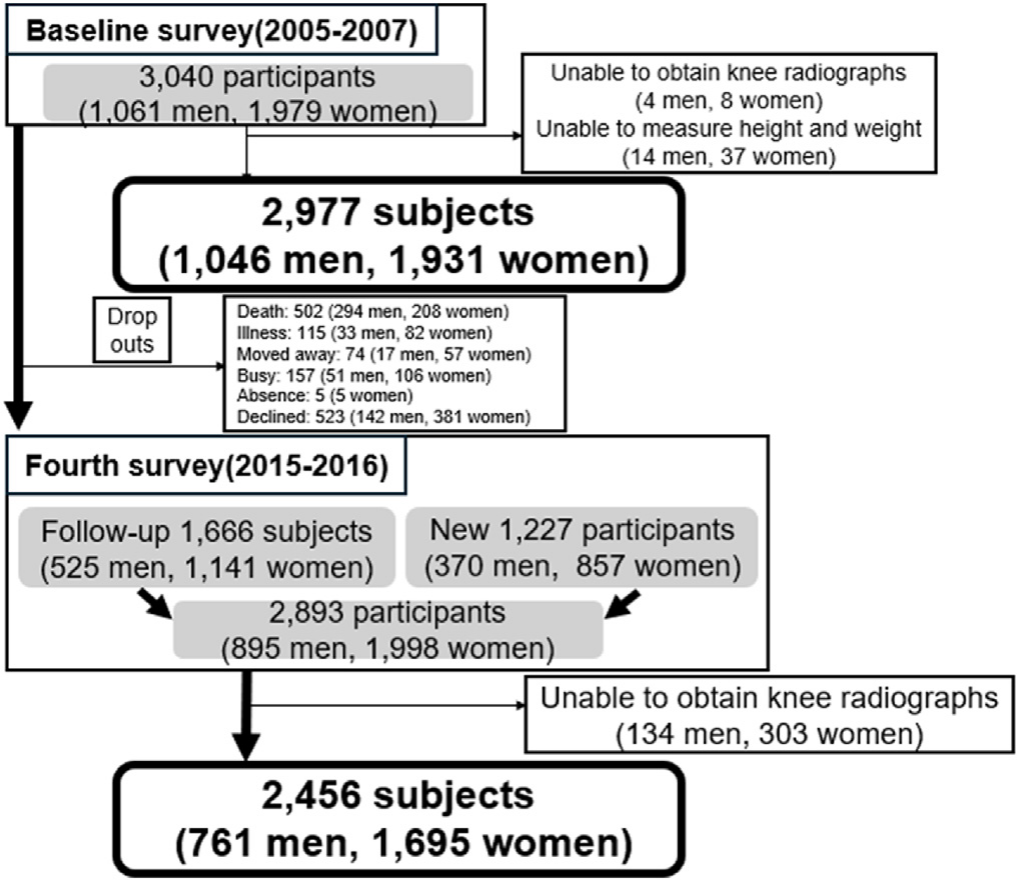
Flow chart of participant selection.

Regarding knee OA, at baseline, 2977 participants (1046 men and 1931 women) fulfilled the inclusion criteria and were enrolled. Notably, the fourth survey was a 10-year follow-up, during which we actively sought a higher number of new participants as volunteers to establish a new baseline by leveraging publications in each respective area. A total of 1666 individuals (525 men and 1141 women) participated in both the baseline and fourth surveys, whereas 1227 individuals (370 men and 857 women) joined as new participants in the fourth survey. The fourth survey included 2893 participants (895 men and 1998 women; mean age, 68.9 years; range, 18–97 years). Consequently, 2456 participants (761 men, 1695 women) met the inclusion criteria (Fig. 1).

Written informed consent was obtained from all participants, and the study was conducted with the approval of the ethics committees of the University of Tokyo and Tokyo Metropolitan Institute of Gerontology.

### 2.2 Examinations performed at each survey

1) Questionnaire

Participants responded to an interviewer-led questionnaire containing more than 400 enquiries covering various aspects, including lifestyle, occupation, smoking and alcohol habits, family and medical history, physical activity, reproductive experiences, and health-related quality of life.

2) Anthropometric measurements

All participants underwent height and weight measurements necessary for calculating the body mass index (BMI) as weight (kg)/height [2] (m^2^).

3) Radiographic assessments

All participants underwent radiographic examination of both knees using an anterior-posterior view with weight bearing and foot map positioning. Fluoroscopic guidance with horizontal anteroposterior X-ray beams ensured proper visualisation of the joint space. Knee radiographs were interpreted by a single experienced orthopaedic surgeon (SM) without knowledge of the participants' clinical status. The Kellgren–Lawrence (KL) grading system was employed, and the KL radiographic atlas was used to assign overall knee radiographic grades [11]. The KL system categorises severity into five grades (0–4), with KL grade 2 indicating osteophyte formation and grade 3 indicating joint space narrowing in addition to osteophyte formation. A KL grade of 2 is commonly regarded as a diagnostic criterion for knee OA. The higher KL grade observed in either knee was considered the participant's grade. Knees that underwent total knee arthroplasty were defined as having a KL grade of 4. To assess the intra- and inter-observer variability of the KL grading, 100 randomly selected radiographs were independently scored by the primary observer and compared with another physician's (HO) ratings, with a repeat evaluation conducted by the same observer after 1 month. Kappa analysis confirmed sufficient agreement for KL grade assessment (0.86 and 0.80, respectively) [9].

4) Symptomatic knee OA assessments

We previously presented data on the prevalence of knee pain and explored its correlation with knee OA at baseline. We defined pain as “a feeling of pain in and around the knee joint at least 1 day during the past 1 month or a complaint of knee pain at physician's examination” [9]. Symptomatic knee OA was defined as the presence of knee OA and pain on the same side. Patients who underwent total knee arthroplasty were excluded because they did not complain of pain. Thus, our objective was to elucidate trends in the prevalence of symptomatic knee OA.

### 2.3 Statistical analysis

Differences in age, height, weight, and BMI between the baseline and fourth surveys were examined using non-paired t-tests. Differences in prevalence between the baseline and fourth surveys were examined using the chi-squared test. The association between the presence of radiographic knee OA in the baseline and fourth surveys and potential associated factors was evaluated using multivariable logistic regression analysis. In the analysis, the absence or presence of radiographic knee OA was used as the objective variable (0 as absent, 1 as present), and the period of survey for the diagnosis of knee OA was used as the explanatory variable (0 as baseline, 1 as fourth survey), after adjustment for age, sex, BMI, and community. Symptomatic knee OA was evaluated using the same method. Data analyses were performed using JMP analyser (version 17.0; SAS Institute, Cary, NC, USA).

## 3 Results

In this study, we analysed the prevalence of radiographic and symptomatic knee OA using data from 2977 participants at baseline (2005–2006) and 2456 participants in the fourth survey (2015–2016). The background characteristics of the participants of the two surveys are presented in Table 1. The mean height of men and women increased over the 10 years. However, an increase in weight was observed only in men, and increased BMI only occurred in women. Significant differences were observed in age, height, and weight in men and age, height, and BMI in women between the baseline and fourth surveys. Residency in urban areas decreased, and that in coastal areas increased.

**Table 1:** Baseline and fourth survey background characteristics of participants.

Number of participants	Overall			Men			Women		
	Baseline	Fourth	p value Baseline vs Fourth survey	Baseline	Fourth	p value Baseline vs Fourth survey	Baseline	Fourth	p value Baseline vs Fourth survey
	2977	2456		1046	761		1931	1695	
Age (years)	70.3 (11.1)	68.9 (13.7)∗∗	<0.0001	71.0 (10.7)	68.4 (14.3)∗∗	<0.0001	69.8 (11.3)	69.1 (13.4)∗	0.028
Height (cm)	154.2 (8.9)	155.8 (9.5)∗∗	<0.0001	162.5 (6.7)	165.5 (7.1)∗∗	<0.0001	149.8 (6.5)	151.5 (6.9)∗∗	<0.0001
Weight (kg)	54.9 (10.3)	55.4 (11.3)	0.1222	61.3 (10.0)	64.0 (11.2)∗∗	<0.0001	51.4 (8.6)	51.5 (9.0)	0.933
BMI (kg/m^2^)	23.0 (3.3)	22.7 (3.5)∗	0.0007	23.2 (3.1)	23.3 (3.3)	0.3699	22.9 (3.5)	22.4 (3.5)∗	<0.0001

Data are means (SD). BMI, body mass index, ∗p ​< ​0.05, ∗∗p ​< ​0.0001 (Baseline vs Fourth survey).

**Fig. 2:**
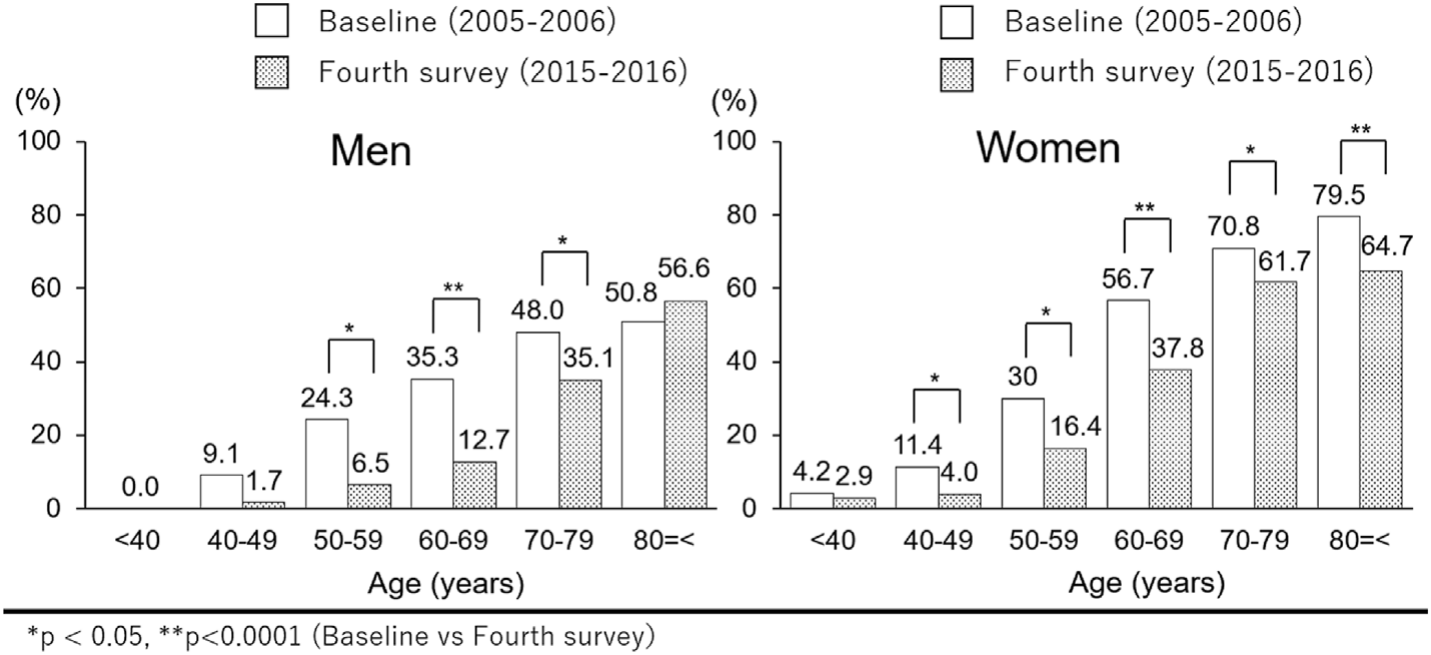
Prevalence (percentage) of radiographic knee osteoarthritis in each age group (<40 years, 40–49, 50–59, 60–69, 70–79, and 80 years or older) at baseline (2005–2007) and fourth survey (2015–2016).

Fig. 2 shows the age-sex distribution of the prevalence of radiographic knee OA at baseline and the fourth survey period. At the baseline survey period, the overall prevalence of radiographic knee OA was 54.2 ​% (men: 42.0 ​%; women: 61.5 ​%). The prevalence in participants <40, 40–49, 50–59, 60–69, 70–79, and ≥80 years was 2.8 ​%, 10.7 ​%, 28.1 ​%, 50.2 ​%, 62.3 ​%, and 68.9 ​%, respectively. At the fourth survey, the overall prevalence of radiographic knee OA was 39.3 ​% (men: 26.9 ​%; women: 44.9 ​%). The prevalence in each age group was 1.7 ​%, 3.2 ​%, 13.3 ​%, 29.5 ​%, 54.7 ​%, and 62.2 ​%, respectively. Graphs were obtained according to sex distribution in each age group. In each group, the prevalence gradually increased, indicating an age-associated increase in OA in both sexes. Significant differences were noticed in men aged 50–59, 60–69, and 70–79 years old and women ≥40 years (Fig. 2).

To evaluate differences in the prevalence of knee OA between the baseline and fourth surveys, we set the absence or presence of knee OA as the objective variable and the timing at which we diagnosed knee OA (baseline or fourth survey) as explanatory variables and conducted a logistic regression analysis after adjusting for age, sex, BMI, and communities. Radiographic knee OA was significantly less prevalent at the fourth survey period compared to the baseline survey period (OR, 0.44; 95%CI, 0.39–0.50; p ​< ​0.0001).

**Fig. 3:**
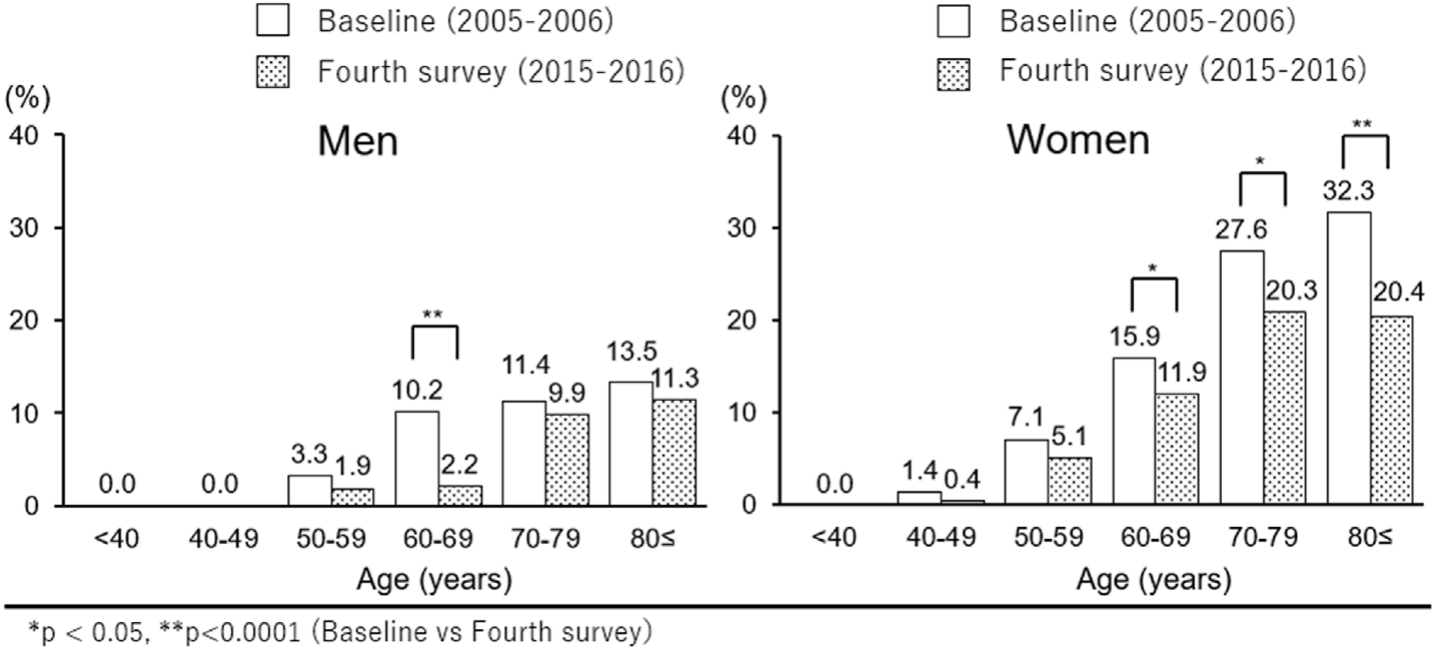
Prevalence (percentage) of symptomatic knee osteoarthritis in each age group (<40 years, 40–49, 50–59, 60–69, 70–79, and 80 years or older) at baseline (2005–2007) and fourth survey (2015–2016).

Regarding symptomatic knee OA, Fig. 3 shows the age-sex distribution of the prevalence of symptomatic knee OA at baseline and the fourth survey period. At the baseline survey, the overall prevalence of symptomatic knee OA was 24.3 ​% (men, 16.9 ​%; women, 28.3 ​%). The prevalence in participants <40, 40–49, 50–59, 60–69, 70–79, and ≥80 years old was 0 ​%, 1.0 ​%, 5.8 ​%, 14.1 ​%, 21.5 ​%, and 25.2 ​%, respectively. At the fourth survey, the overall prevalence of symptomatic knee OA was 20.6 ​% (men: 14.2 ​%; women: 23.5 ​%). The prevalence in each age group was 0 ​%, 0.3 ​%, 4.1 ​%, 8.6 ​%, 17.6 ​%, and 17.5 ​%, respectively. Graphs were obtained according to sex distribution in each age group (Fig. 3).

We evaluated the differences using the same method as for radiographic knee OA and conducted a logistic regression analysis. Symptomatic knee OA significantly reduced over the 10-year period (OR, 0.54; 95%CI, 0.48–0.61; p ​< ​0.0001).

## 4 Discussion

This study clarified the prevalence of knee OA in three cities using data obtained at the baseline and the fourth survey of the ROAD study which was conducted 10 years after the initial survey. The prevalence of knee OA at the fourth survey was 39.3 ​% in the overall population. Comparing these results with the baseline survey data, in which the prevalence of knee OA was 54.2 ​% in the overall population revealed that the prevalence had declined in those 10 years (p ​< ​0.0001). We observed a similar tendency for symptomatic knee OA as that of radiographic knee OA. At the baseline survey, the overall prevalence of symptomatic knee OA was 24.3 ​%, whereas at the fourth survey, it was 20.6 ​%; a significant reduction was confirmed (p ​< ​0.0001).

The present study clarified the age-sex distribution of the prevalence of knee OA diagnosed radiographically in Japanese populations. When we applied the results obtained from the baseline survey of the ROAD study to the total age-sex distribution derived from the Japanese census in 2005 [12], we estimated that 25,300,000 people (8,600,000 men and 16,700,000 women) aged ≥40 years would be affected by radiographic knee OA. Asymptomatic patients with OA were included in this study. Moreover, one-quarter of men with radiographic OA and one-third of women with radiographic OA were reported to have pain, which is considered symptomatic OA [13,14]; therefore, approximately 7,800,000 people (2,200,000 men and 5,600,000 women) aged ≥40 years were estimated to be affected by symptomatic knee OA. However, when we applied the results obtained from the fourth survey of the ROAD study to the total age-sex distribution derived from the Japanese census in 2015 [15], we estimated that 20,000,000 people (6,000,000 men and 14,000,000 women) aged ≥40 years would be affected by radiographic knee OA. Moreover, according to our results, approximately 5,800,000 people (1,400,000 men and 4,400,000 women) aged ≥40 years were estimated to be affected by symptomatic knee OA.

To explain this trend, we made assumptions about the cause of this decline. First, the rejuvenation of the overall population may have occurred. Similar results were observed for the morphological structures of knee OA. Kitamura et al. compared the medial minimum joint space width of bilateral knees in the baseline and fourth surveys of the ROAD study [16]. Using knee OA computer-aided diagnosis systems, they found that the minimum joint space width broadened over 10 years and suggested that their results may be due to rejuvenation in the overall population. This broadening phenomenon correlates with a decline in knee OA. In our study, we evaluated knee OA using the KL grading system, and our trend matched the results, possibly indicating that rejuvenation may have occurred over 10 years.

In the ROAD study, we previously clarified the tendency of the prevalence of osteoporosis to decline between 2005 and 2015 [7]. At the baseline survey, the prevalence of lumber spine (L2–L4) osteoporosis was 13.6 ​% (men, 3.4 ​%; women, 19.2 ​%); at the fourth survey, it was 9.7 ​% (men, 1.4 ​%; women 13.9 ​%), with a significant decrease (p ​< ​0.0001). We assumed that 1) better nutritional intake, especially in calcium, 2) the positive effect of prevention measures conducted by the government and academic societies, and 3) multiple participation in the ROAD study, which led to the acquisition of medical advice, were the leading causes of the decline in prevalence. Moreover, we have revealed the trend in the prevalence of hip OA between 2005 and 2015; the logistic regression analysis revealed that the prevalence of radiographic hip OA was significantly lower at the fourth survey than at the baseline survey (OR, 0.55; 95%CI, 0.46–0.65) [8]. We found that increased hand grip strength correlates with a decreased prevalence of hip OA. In this study, hand grip strength was strongly associated with lower extremity muscle strength, especially the quadriceps; we concluded that the prevalence of hip OA declined due to the increase in muscle strength throughout the body. These results also suggest that the whole population underwent rejuvenation during the 10 years; we also noticed that the trend of rejuvenation is consistent with that of the bones and joints.

Regarding knee OA, similar correspondence can be assumed for knee OA. Previous studies have reported that a decrease in muscle strength is an important factor for the formation of knee OA [17]. Gong et al. obtained data from a longitudinal observational study of 1338 patients (523 men, 815 women, aged 45–79 years; mean age 61.8 years) and found that an increase in lower extremity muscle strength, especially in the quadriceps, prevents cartilage degeneration and synovitis, leading to the progression of knee OA [18]. Lihui et al. evaluated knee OA and hand grip strength and found that handgrip strength negatively affected hand and knee OA in men and women (p ​< ​0.05) [19]. Other reports support the correlation between hand grip strength and lower extremity muscle strength [[20], [21], [22]]. These results strongly indicate the quadriceps, one of the key muscles for prevention of knee OA, are associated with handgrip strength. Therefore, the overall increase in hand grip [8] leads to the assumption that an increase in quadriceps strength may occur simultaneously, which may have influenced the decrease in the prevalence of knee OA. These factors suggest that people in recent generations have increased overall muscle strength, and strengthening the muscles around the knee joints may be a factor for the decrease in the prevalence of knee OA. Because we have yet to analyse the relationship between muscle strength and mass, further confirmation is necessary.

We investigated whether this declining trend was relevant to anthropometric measurements and physical activity. According to the Ministry of Health, Labor, and Welfare, the mean height of men and women in 2005 was 166.6 ​cm (standard deviation 7.2 ​cm) and 153.4 ​cm (6.9 ​cm), respectively [23]; in 2015, the mean height was 167.5 ​cm (7.1 ​cm) and 154.2 ​cm (7.0 ​cm), respectively [24]. In the overall Japanese population, we observed a 1 ​cm increase in height over 10 years. In our study, we observed the same trend in height for men and women (Table 1). Geoffrey et al. used novel fast-acquisition MRI to quantify the volumes and lengths of 35 major lower limb muscles in 24 young, healthy participants and determine whether muscle size correlates with bone geometry and subject parameters of mass and height [25]. They found that total lower limb muscle volume scales with mass and the height–mass product. This indicates that a greater height is equivalent to greater muscle mass. Hence, we can assume that an increase in height may be relevant to gaining muscle strength and mass, which may have contributed to the declining trend in knee OA over 10 years.

Another possibility is that better nutritional intake during adolescence may have influenced rejuvenation. We previously mentioned that nutritional intake during the adolescent period may result in better results in terms of osteoporosis [7]. However, regarding knee OA, we assume that increasing the intake of protein may have a positive effect on reducing knee OA. Protein is a nutrient essential for energy production; it constitutes a significant portion of skeletal muscle in organisms and plays a key role in regulating metabolism [26]. A decrease in muscle mass may be accelerated by a decline in the assimilation response to insufficient protein intake [27]. Tagawa et al. studied 5402 participants (age range 19–81; mean age 47.2 years) from 105 articles to evaluate the dose-response relationship of the effects of protein intake on lean body mass [28]. They found that even a slight increase in protein intake by 0.1 ​g/kg of body weight per day for several months in a dose-dependent manner over a range of doses from 0.5 to 3.5 ​g/kg of body weight per day may increase or maintain body mass, which suggests a positive correlation between protein intake and body mass. It is commonly considered that during adolescence or teenage years, the growth rate is at its maximum due to the strong effect of nutrition intake. Participants in their 50s and 60s in 2005 and 2015 were adolescents in 1961 and 1971, respectively. According to the Ministry of Health, Labor, and Welfare, in the 1971 National Nutrition Survey of Japan, the daily intake of total protein increased from 70.0 ​g in 1961 to 78.1 ​g in 1971 [29]. On the contrary, no increase was found in total protein intake between 2005 and 2015 [30,31]. Therefore, it is estimated that protein increase had affected the lower extremity muscle mass and strengthened the knee joints. Further investigation is needed to consider other factors contributing to the decrease in prevalence.

Other studies in Japan supports our findings as well. Using the cohort data obtained from 2007 to 2017, Suzuki et al. observed an increase in overall height, walking speed, and hand grip strength and a decrease in BMI in women (p ​< ​0.05); they concluded that better nutrition intake, faster walking speed, and lower BMI (or a desire to be “slim”) are key factors that invoked functional improvement of the human body over the previous few decades [32]. They suggested that the increased number of “young-old people” may explain the rejuvenation of health status. These findings indicate that rejuvenation occurs in younger populations through rejuvenation effects.

This study has some limitations. First, a selection bias regarding the percentage of participants in their residences may have influenced the results. Specifically, the number of participants from coastal areas decreased, while the number of participants from coastal areas increased. This shift may serve as a source of sampling bias. There are significant differences in age, height, and BMI between the participants of the baseline and the fourth survey (Table 1). Although these factors were adjusted for in the analysis, when interpreting the results, we must consider that the fourth survey population tends to be relatively younger and have lower BMI, and there is a possibility that these regional differences are affecting the results. We have yet to obtain enough participants aged ≤40 years old, and the knee OA prevalence in younger generations may be different from that reported.

Second, it is worth noting that both the baseline and the new participants might have higher health awareness, which could introduce selection bias. However, the lack of overlap among participants in the same age group led us to determine that the KOA imaging results are comparable. Nevertheless, since the study employs a method of comparing cross-sectional surveys within a cohort study, there remains a possibility that selection bias has not been completely eliminated.

Third, this study included participants living independently instead of those living in institutional settings. Therefore, the calculated prevalence of radiographic knee OA may have been underestimated. Although the ROAD study included a large cohort, the participants in the present study may not be representative of the general Japanese population. In an earlier study, we compared the anthropometric measurements and lifestyle factors, such as smoking and drinking habits, between the study participants of the baseline survey of the ROAD study and the general Japanese population [33]. We found no significant differences between the two, except for the lower proportion of current smokers and drinkers in our study population than in the general Japanese population, suggesting that our study participants led healthier lifestyles. This selection bias should be taken into consideration when generalizing the results obtained from the present study.

In conclusion, we compared the prevalence of knee OA in three communities at baseline and the fourth survey of the ROAD study over a 10-year period and found that the prevalence of knee OA declined over 10 years, and rejuvenation of bone and joint health may occur in all populations. It is vital to continue this longitudinal study to contribute to the knowledge and prevention of knee OA.

## Author contributions

All authors made substantial contributions to all three of the following: (1) conception and design of the study, acquisition of data, or analysis and interpretation of data. (2) Drafting the article or revising it critically for important intellectual content and (3) final approval of the version to be submitted.

## Declaration of generative AI in scientific writing

No generative AI in this scientific writing was used.

## Role of the funding source

This work was supported by a Grant-in-Aid funding from the Ministry of Health, Labour and Welfare: H17-Men-eki-009 (Director, Kozo Nakamura), H23-Choujyu-002 (Director, Toru Akune), H20-Choujyu-009, H25-Choujyu-007 (Director, Noriko Yoshimura), H25-Nanchitou (Men)-005 (Director, Sakae Tanaka), 24FA1006 (Director, Noriko Yoshimura), 19FA1401, 22FA1009, 24FA1003 (Director, Sakae Tanaka), 19FA1007, 20JA1001 (Director, Hiroyuki Oka), 19FA1017 (Director, Etsuo Chosa), 19FB1001 (Director, Yutaka Osuga), and 21FA1006 (Director, Hiroshi Yamada). The study was also supported by Scientific Research grants B19H03895, B26293139, B23390172, and B20390182, and Challenging Exploratory Research grants 21K19631, 18K18447, 15K15219, and 24659317 to Noriko Yoshimura; Scientific Research grants B26293331, B23390356, and C20591774 and Challenging Exploratory Research grants 26670307 and 23659580 to Shigeyuki Muraki; Challenging Exploratory Research grants 24659666 and 21659349 and Young Scientists A18689031 to Hiroyuki Oka; Scientific Research grants B26293329, B23390357, and C20591737 and Challenging Exploratory Research grant 25670293 to Toru Akune; Scientific Research grant S50282661 to Sakae Tanaka; and by Collaborating Research with NSF from the Ministry of Education, Culture, Sports, Science and Technology in Japan 08033011–00262 (Director, Noriko Yoshimura). This study was partly supported by grants from the Japan Agency for Medical Research and Development (17dk0110028h0001, Director, Noriko Yoshimura; 17gk0210007h0003, 19gk0210018h0002, 22gk0210034h0001, and 23gk0210034h0002, Director, Sakae Tanaka; 22dk0110047h0001, Director, Kanae Mure; 22dk0110048h0001, Director, Hiroyuki Oka; @@, Director, Akiko Kishi). Furthermore, the study was partly supported by grants from the Japan Osteoporosis Society (Noriko Yoshimura, Chiaki Horii, Shigeyuki Muraki, Hiroyuki Oka, and Toru Akune) and Japan Osteoporosis Foundation (2015, Noriko Yoshimura, and 2019, Toshiko Iidaka) and research aid from the Japanese Orthopedic Association (JOA-Subsidized Science Project Research 2006-1 and 2010-2, Director, Hiroshi Kawaguchi; and 2014-1, Director, Kozo Nakamura), the Japanese Society for Musculoskeletal Medicine (2015, Director, Shigeyuki Muraki; and 2017, Director, Noriko Yoshimura), Foundation (2016, Director, Noriko Yoshimura), Association (2017, Director, Noriko Yoshimura).

## Declaration of competing interest

There are no conflicts of interest.
